# Loss of STAT2 may be dangerous in a world filled with viruses

**DOI:** 10.1172/JCI170886

**Published:** 2023-06-15

**Authors:** Michael B. Jordan

**Affiliations:** Divisions of Immunobiology and Bone Marrow Transplant and Immune Deficiency, Cincinnati Children’s Hospital Medical Center, University of Cincinnati Medical School, Cincinnati, Ohio, USA.

## Abstract

Type I IFNs, a family of cytokines that signal through a single receptor and signaling mechanism, were originally named for their ability to interfere with viral replication. While type II IFN (IFN-γ) largely protects against intracellular bacteria and protozoa, type I IFNs largely protect from viral infections. Inborn errors of immunity in humans have demonstrated this point and its clinical relevance with increasing clarity. In this issue of the *JCI*, Bucciol, Moens, et al. report the largest series of patients to date with deficiency of STAT2, an important protein for type I IFN signaling. Individuals with STAT2 loss demonstrated a clinical phenotype of viral susceptibility and inflammatory complications, many of which remain poorly understood. These findings further illustrate the very specific and critical role that type I IFNs play in host defense against viruses.

## IFN signaling and resistance to viral infections

**Figure 1:**
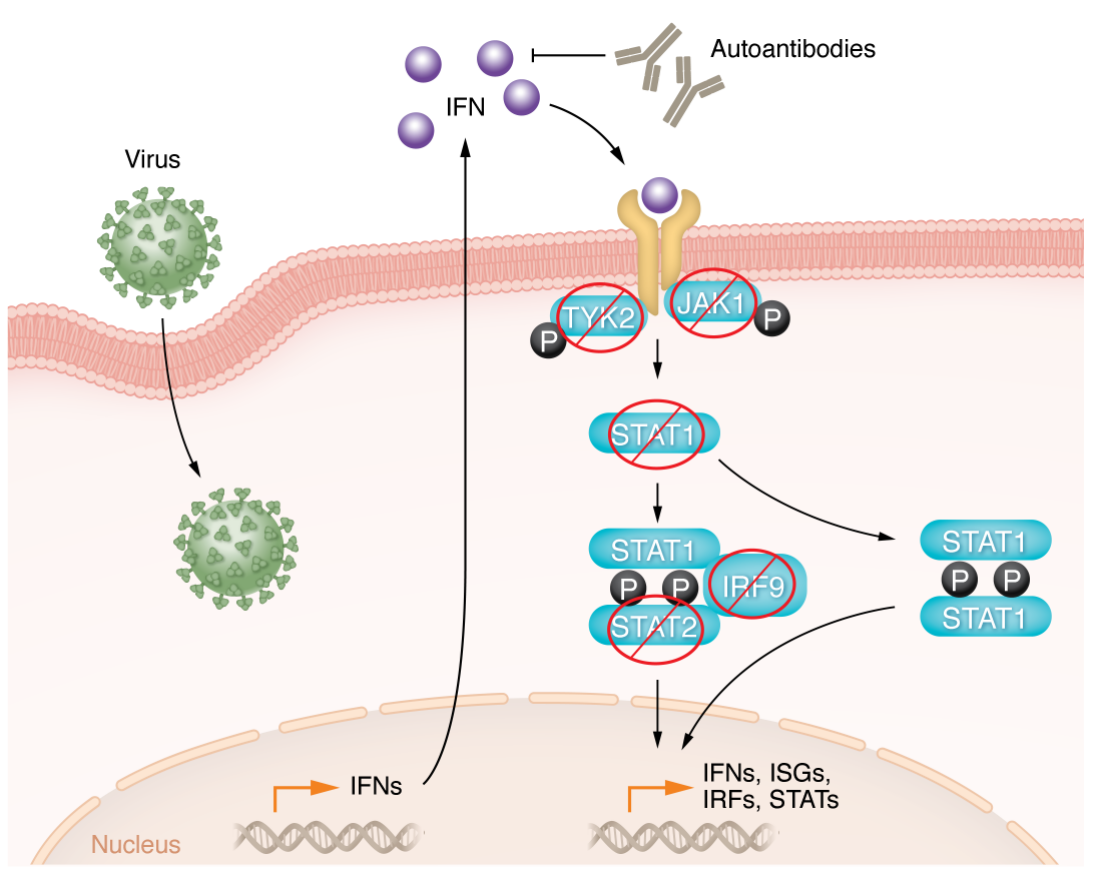
STAT2 deficiency and other inborn and acquired defects of type I IFN signaling result in susceptibility to viral infections. Inborn errors of immunity affecting molecules important for type I IFN signaling, including IFNAR1, IFNAR2, TYK2, JAK1, STAT1, and IRF9, have been previously reported to cause phenotypes of severe viral infection. Bucciol, Moens, et al. (1) now report on a large series of patients with STAT2 deficiency, indicating that STAT2 loss can lead to pronounced complications with viral infections. Type I IFNs, including multiple α and β IFNs, signal through the type I IFN receptor, which utilizes the JAK/STAT signaling pathway. In addition, acquired autoantibodies against type I IFNs have been described in many patients with severe SARS-CoV-2 infection. These manifestations indicate that type I IFNs are a critical defense against frequent viral exposure. ISG, IFN-stimulated gene; PRR, pattern recognition receptor.

In this issue of the *JCI*, Bucciol, Moens, et al. describe the phenotypes of an extended cohort of patients with STAT2 deficiency, extending prior reports of individual patients or families and giving a more complete picture of human STAT2 deficiency (1). In this report, 23 patients from 10 families are described with biallelic loss-of-function variants in STAT2 and who predominantly suffered from difficulty in controlling viruses. Other inborn genetic errors affecting the type I IFN–signaling pathway, including gene defects in IFNAR1, IFNAR2, and IRF9, have previously been described and cause similar difficulties with wild viruses and live attenuated viral vaccines (1–4). Loss-of-function variants in STAT1 and partial loss-of-function of JAK1 have also been reported as causing susceptibility to viruses, but befitting their dual role in type I and type II (IFN-γ) IFN signaling, patients with these variants experience greater difficulties with mycobacterial infections (5, 6). Thus, inborn genetic errors in essentially the entire proximal type I IFN-signaling pathway (Figure 1) have demonstrated the importance of this pathway for resistance to a variety of viruses. As is noted by Bucciol, Moens, et al., STAT2 and related genes are under intense selective pressure, presumably driven by viruses. In recent years, it has also become increasingly appreciated that various impairments of type I IFN function, either from inborn errors or anti-IFN autoantibodies, have played an important role in morbidity and mortality from the SARS-CoV-2 pandemic (7, 8). Thus, increasing evidence demonstrates that type I IFNs are a critical defense against the ubiquitous threat that viruses pose.

## Impact of STAT2 deficiency on viral infections

A majority of the patients studied in Bucciol, Moens, et al. experienced clinical complications after receiving live attenuated viral vaccines. Indeed, almost 70% of those receiving live viral vaccines, including measles-mumps-rubella (MMR) and varicella, experienced complications, such as prolonged febrile illness, encephalitis, other organ injury, atypical Kawasaki disease, and hemophagocytic lymphohistiocytosis. Naturally occurring viral infections, including influenza, enterovirus, herpes simplex, and SARS-CoV-2, also caused serious infection in a substantial fraction of these patients. Notably, though, a number of naturally occurring viral infections, including SARS-CoV-2, were experienced by individuals in this cohort without complications. Bacterial infections were also noted in some cases, principally pneumonias. Approximately 40% of the patients in this series died prematurely. Most deaths were during early childhood, with none reported after approximately four years of age. This age distribution of mortality reminds one that the immune system undergoes intense virus-driven education in the first years of life and suggests that this education eventually provided protection even with defective type I IFN signaling. Indeed, intravenous immunoglobulins (IVIG) appeared to be therapeutic in some cases in the current report (1).

## Unexplained inflammatory complications

In addition to severe infection, a number of inflammatory complications were notable in this cohort. Atypical Kawasaki disease was noted in six patients. Cytopenias and other clinical features consistent with secondary hemophagocytic lymphohistiocytosis were observed in two patients, while transient cytopenias were observed in five. Remarkably, six patients died of heart failure in the context of febrile illness without identified pathogens. It is unclear whether these deaths were due to viral myocarditis or other unknown and/or inflammatory etiologies. Overall, it is unclear whether these inflammatory complications were entirely driven by abnormally severe or persistent viral infection (as viruses were not always identified) or were due to an intrinsic immune dysregulation. Indeed, there is experimental evidence to suggest that loss of STAT2 may lead to aberrant inflammatory signaling in macrophages, which is more type II IFN–like (9). There is still much to learn regarding the pro- and antiinflammatory effects of the IFNs and how these effects may be helpful or maladaptive in specific contexts.

## Lessons regarding care for STAT2-deficient individuals

Bucciol, Moens, et al. provide the most complete picture of the clinical spectrum of STAT2 deficiency to date. Treatment in this cohort included high-dose IVIG and antiviral agents as either standard treatment or prophylaxis. The high mortality of this cohort suggests that STAT2 deficiency may be appropriately treated with bone marrow transplantation. However, the curative potential for STAT2 deficiency has yet to be demonstrated with this risky modality. Indeed, the lack of late deaths in this cohort suggests that mortality risk after early childhood may be low. Thus, it is difficult to weigh the risks and benefits of transplantation. Despite these caveats, this series suggests that one could reasonably consider proceeding to allogeneic transplantation with the identification of a very young presymptomatic patient (likely a sibling of a seriously affected child) who has a well-matched donor graft available. In the future, with the likely widespread preemptive use of genetic testing, determining the potential impact of rare mutations in genes such as STAT2 will be of increasing importance for patients and the practice of medicine.

## IFNs are our friends in a hostile world

In a potentially hostile world with innumerable viruses and ubiquitous soil and waterborne bacteria with pathogenic potential, type I and type II IFNs play an essential protective role. Inborn errors of immunity as well as acquired impairments, such as development of anticytokine antibodies, have demonstrated that type II IFN is critical for defense against mycobacteria and other intracellular bacterial and protozoan pathogens, while type I IFNs play a critical role in host defense against viruses. The function of type III IFNs, which also rely substantially on STAT2 for signaling, is less clear in humans, but likely overlaps with that of type I IFNs. Increasing hygiene and antimicrobial drugs in the modern world may have deceased the need to protect from various bacterial pathogens, but viruses remain an ever-present threat and type I IFNs are increasingly appreciated as an essential part of our defense.

## Version 1 06/15/2023

Electronic publication
